# Using Dose–Response Correlation Re-Analyzing to Distinguish Placebo from Standardized Rose-Hip Powder (Lito) in a Clinical Trial on Osteoarthritis Where Data Initially Looked Identical

**DOI:** 10.3390/nu18020331

**Published:** 2026-01-20

**Authors:** Alzahraa Mahmoud Motawei, Kristian Marstrand Warholm, Kaj Winther

**Affiliations:** 1 Food Sciences, Faculty of Agriculture, Mansoura University, Mansoura 35516, Egypt; elzahraa@mans.edu.eg; 2 Division of Orthopedic Surgery, Oslo University Hospital, 0586 Oslo, Norway; 3 Department of Nutrition, Exercise and Sports, University of Copenhagen, 1958 Frederiksberg C, Denmark

**Keywords:** placebo, correlation-analyzing, dose-response

## Abstract

Background: Large positive responses to placebo are common in clinical trials and pose a major challenge when evaluating different treatments, including new foods. Standard between-group comparisons may fail to detect true effects when placebo improvements are significant. We aimed to demonstrate how a simple dose–response correlation method can help differentiate genuine positive responses from those experienced with placebo through secondary analysis of a randomized controlled clinical trial of powdered *Rosa-canina* fruits. Methods: Data were reanalyzed from a multicenter, double-blind, randomized, placebo-controlled trial (N = 120; ClinicalTrials.gov NCT01459939) evaluating the effects of standardized *Rosa-canina* powder in hip and knee osteoarthritis (OA). Participants received fixed doses, leading to variability in mg/kg exposure due to different body weights. Pearson correlations between dose/kg and changes in WOMAC pain and function at 6 and 12 weeks were calculated separately for the active and placebo groups. Standard between-group comparisons were also performed. Results: Both groups showed significant improvement, over 50%, with no statistically significant differences between them in WOMAC pain or function. However, only the active group, which received a food supplement, exhibited a consistent negative correlation between body weight and symptom improvement at 6 and 12 weeks, suggesting greater benefit with higher dose per kilogram of body weight. No dose–response relationship was observed in the placebo recipients. Therefore, weight-stratified plots revealed an exposure–response gradient in the active group. Conclusions: Dose–response correlation analysis uncovered positive effects of *Rosa-canina* as a nutrient that were not detectable through standard between-group comparisons. This is consistent with findings from earlier rose-hip research. This low-cost, easy-to-implement method may help distinguish active effects from placebo responses in trials with large nonspecific improvements. Incorporating such analyses could improve the identification of nutrients containing biologically active preparations and support dose selection in future clinical research.

## 1. Introduction

Osteoarthritis (OA) is a progressive degenerative joint disease associated with chronic pain, stiffness, functional limitations, and reduced quality of life. Many patients turn to botanical or nutraceutical therapies such as glucosamine, chondroitin sulfate, turmeric extracts, and *Rosa-canina* for symptom relief. However, major randomized trials and meta-analyses have reported inconsistent or null findings for widely used nutraceuticals, including glucosamine and chondroitin [1,2,3].

A major methodological challenge in assessing such interventions is the high level of placebo responses in pain and activity of daily living, which can exceed 60% [1]. These substantial nonspecific effects can hide actual therapeutic benefits and make trial results harder to interpret [1,2,3,4,5,6,7,8,9,10,11,12,13,14,15,16].

Subspecies Lito from *Rosa-canina* L., Rosaceae (rose-hip) has demonstrated analgesic and anti-inflammatory potential in several human trials [5,6,7], although results vary across studies. Differences in formulation, bioactive content, and dosing may contribute to inconsistent outcomes [17,18,19]. Importantly, most botanical products are administered at a fixed daily dose, despite significant variation in patient body weight. This results in natural variability in mg/kg exposure, offering a valuable opportunity to examine whether symptom improvement correlates with effective dose—a key expectation of true pharmacologic effects [14].

We therefore propose a simple analytic method: evaluating correlations between body-weight-adjusted dose and clinical improvement. If an intervention exerts pharmacologic effects, greater dose/kg should be associated with greater improvement. If improvements arise solely from placebo mechanisms, no such relationship should be present.

Using secondary analysis of a randomized, placebo-controlled trial of *Rosa-canina* [8,10], we illustrate how this approach can reveal pharmacologic signals otherwise obscured by large placebo responses. The study is supported by two other studies using the same subspecies of rose hip [10,12].

## 2. Methods

### 2.1. Study Design and Participants

This study is a secondary analysis of data collected from an investigator-initiated, multicenter, double-blind, randomized, placebo-controlled, phase III parallel-group trial assessing a standardized *Rosa canina* powder in patients with mild to moderate osteoarthritis (OA) of the hip and/or knee (total n = 120; active treatment n = 60, placebo n = 60) [8].

**Figure 1:**
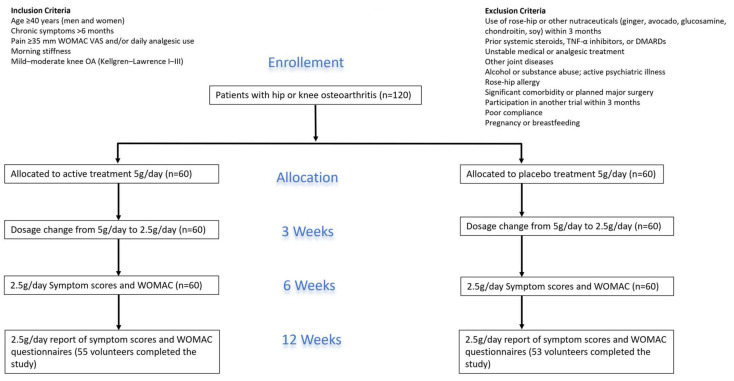
Flow Chart.

Participants were recruited through notices in local newspapers. They were eligible if they were men or women aged 40 to 80 years with a clinical diagnosis of hip or knee OA based on the American College of Rheumatology criteria, including history and physical examination (details in flow chart—Figure 1). Exclusion criteria included inflammatory rheumatic diseases, joint disorders other than OA, recent intra-articular corticosteroid injections, and known hypersensitivity to rose-hip products. Patients were also excluded if they had used rose-hip powder, avocado–soybean unsaponifiables, ginger, glucosamine, or chondroitin sulfate within the three months prior to screening.

Additional exclusion criteria included treatment with systemic corticosteroids, tumor necrosis factor-α inhibitors, or disease-modifying antirheumatic drugs in the months prior to enrollment; substance abuse; psychiatric illness; planned major joint surgery; participation in another clinical trial within the past three months; or any condition likely to impair study compliance, including alcohol and drug abuse. Volunteers in stable treatment for diabetes and/or cardiovascular disease were allowed to participate. Use of analgesics such as nonsteroidal anti-inflammatory drugs, paracetamol, codeine, or tramadol was permitted only if patients had been on a stable dose for at least 14 days before starting the study and maintained that dose throughout the 12-week period.

The original trial protocol was approved by the local Ethics Committee (Case no. M-20110185), the Danish Data Protection Agency (J. No. 2011-41-6721), and registered at ClinicalTrials.gov (Identifier: NCT01459939). All participants provided written informed consent before enrollment.

### 2.2. Intervention

Participants were randomly assigned to receive either a standardized *Rosa canina* powder or a matched placebo, both administered orally in identical capsules (details in flow chart). Randomization was carried out in blocks of 20 using a computer-generated allocation sequence. All study products were produced from a single batch to ensure consistency.

The rose-hip preparation, produced by Hyben-Vital (Langeland, Denmark), consisted of dried whole fruits from selected *Rosa canina* subtypes containing equal parts shells and seeds. The product was processed using a patented drying method in which seeds and flesh are dried separately and then combined, with the drying temperature never exceeding 39 degrees Celsius. This methodology also ensured that all itchy hairs on the seeds were removed [8]. Placebo capsules and the capsules containing rose-hip powder were produced by a company licensed to produce capsules for clinical trials: Bifodan (Deerland), Bogbindervej 6, 3390 Hundested, Denmark. All capsules contained 0.5 g of either placebo or rose-hip powder and were identical in appearance, taste, smell, and color.

The dosing regimen involved taking 5 g/day (10 capsules) for the first three weeks, then reducing to 2.5 g/day (5 capsules) for the remaining nine weeks, for a total treatment duration of 12 weeks. Treatment adherence was monitored through capsule counts after 3 weeks and 3 months. This indicates that the lightest volunteer (55 kg) received about 910 mg/kg body weight in the first three weeks and about 455 mg/kg body weight in the following 9 weeks. This should be compared with the heaviest volunteer (120 kg), who received about 416 mg/kg bodyweight in the first 3 weeks, followed by about 208 mg/kg bodyweight in the remaining 9 weeks of the study. The patients were instructed to track their intake throughout the study. They were not allowed to start new analgesic treatments or change their diet or physical activity habits during the trial.

### 2.3. Outcomes

For this secondary analysis, the main outcomes were changes in WOMAC pain and WOMAC physical function (Activities of Daily Living) scores at 6 and 12 weeks. WOMAC is a validated, disease-specific tool commonly used to evaluate pain and functional impairment in patients with osteoarthritis [9]. The WOMAC questionnaire uses visual analogue scales and consists of five questions on pain, two questions on stiffness, and seventeen questions on daily activities. The mean of the five questions on pain, the two questions on stiffness, and the seventeen questions on daily activities are calculated separately. The overall WOMAC score is obtained by adding the mean values of pain, stiffness, and daily activities.

#### Dose–Response Correlation Analysis

**Figure 2:**
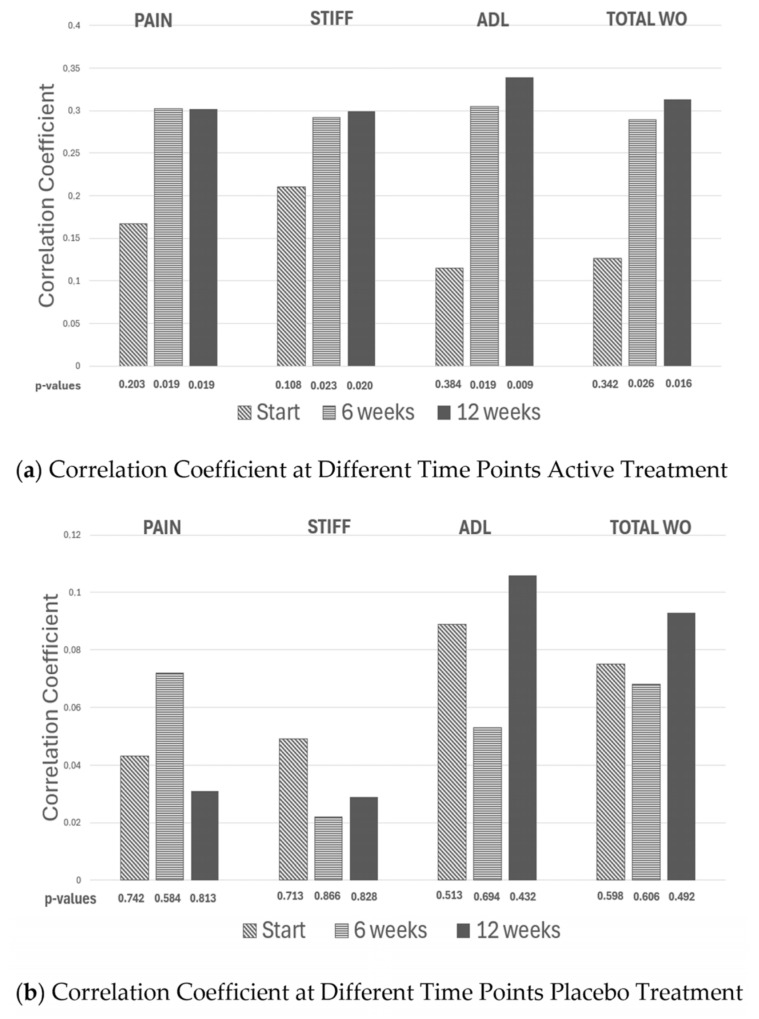
The correlation coefficients generated from plotting weight vs. the delta change in WOMAC pain, WOMAC stiffness, WOMAC Activity of daily living (ADL) and total WOMAC (the different WOMAC scores added together) is given initially and after 6 and 12 weeks of active (**a panel**) and placebo treatment (**b panel**), respectively.

Since all participants consumed a fixed daily amount of either *Rosa-canina* or a placebo, the actual dose per kilogram naturally varied with individual body weight. This variation enabled the investigation of whether clinical improvement increased with exposure. For both the active and placebo groups, at 6- and 12-week assessments, Pearson correlation coefficients were calculated between dose per kilogram and changes in WOMAC pain, stiffness, and physical function, as well as the overall WOMAC score. A negative correlation was interpreted as evidence that a higher dose per kilogram was associated with greater symptom improvement. For details, see Figure 2a,b.

### 2.4. Statistical Analysis

Within-group changes were analyzed using Wilcoxon signed-rank tests. Between-group differences were evaluated with Mann–Whitney U tests. Pearson correlation coefficients (r) and their corresponding *p*-values were reported for dose–response analyses. A two-sided *p* < 0.05 was considered statistically significant.

## 3. Results

### 3.1. Participant Characteristics

A total of 120 participants were randomized (active n = 60; placebo n = 60). Baseline demographics were similar between groups. The mean age was 62.7 ± 8.9 years in the active group and 64.5 ± 10.3 years in the placebo group; 57% and 62% were women, respectively.

### 3.2. Clinical Outcomes

Both groups showed significant within-group improvements in WOMAC pain and function at 6 and 12 weeks, consistent with well-established placebo responses in OA trials [1,15]. After 12 weeks, the average pain improvement was −1.41 ± 7.71 in the active group and −3.55 ± 6.98 in the placebo group; the difference between the groups was not statistically significant, but close to (*p* = 0.058). Similar trends were seen for WOMAC function.

These findings replicate the original trial results: large placebo responses mask between-group differences despite meaningful within-group improvements.

### 3.3. Dose–Response Correlation

No relationship was observed between dose per kilogram and symptom improvement in the placebo group (all *p* > 0.40). In contrast, the active group showed consistent negative correlations between body weight and improvement across WOMAC domains at both time points (e.g., pain r ≈ −0.30; *p* ≈ 0.02), indicating greater improvement with higher exposure—an expected property of pharmacologic treatments [14].

Aggregated WOMAC scores showed the same pattern: greater improvement was observed in participants receiving a higher effective dose per kg—and again—no such pattern was observed in the placebo group.

### 3.4. Weight-Stratification

Weight-stratified analyses indicated that participants weighing over 84 kg showed minimal or no improvement in the active group, while those with lower body weight improved significantly more. No similar pattern was observed in the placebo group, supporting the idea that the dose–response relationship is due to pharmacologic exposure rather than nonspecific placebo effects.

### 3.5. Weight Stratification in Two Other Studies

The present observations are supported by another study on osteoarthritis using the same rose-hip subspecies [10]. Comparing groups revealed statistically significant differences in pain. However, weight stratification applied to the placebo group did not show any correlation. Conversely, when analyzing the actively treated group, a significant negative correlation was observed after three weeks and after three months (*p* < 0.019 and *p* < 0.014), respectively. In another placebo-controlled clinical study, this time on rheumatoid arthritis [12], there was no statistically significant difference in the HAQ pain score between the placebo and actively treated groups. Again, when weight stratification was applied to the two groups, a significantly negative correlation was found between weight and reduction in pain score in the actively treated group (correlation coefficient −0.376, *p* = 0.012 and correlation coefficient −0.321, *p* < 0.033) after three and six months of treatment, respectively. In the placebo group, no such correlation existed.

## 4. Discussion

Traditional between-group statistical comparisons were insufficient to differentiate the effects of *Rosa-canina* from the significant placebo responses seen in this study. Possibly, this is not so surprising as placebo improvements in OA often exceed 50% in validated pain and function scores [1]. Under such conditions, small to moderate pharmacologic effects of botanicals are easily hidden.

When variability in exposure was considered by analyzing body-weight–adjusted dose, a clear and consistent dose–response pattern appeared in the actively treated group but not in the placebo group. Higher effective doses per kilogram were linked to greater symptom improvement, while placebo recipients showed no such relationship. The present observations were supported by two other studies, one on osteoarthritis [10] and one on rheumatoid arthritis [12] in which weight stratification sub analyses were performed and improved our understanding of data. These observations highlight a key methodological challenge in clinical research: most products are given at fixed daily doses despite significant differences among individuals in body size, metabolic rate, and absorption. Some participants may effectively be “under-dosed”, especially heavier individuals, while lighter participants might inadvertently receive an exposure above the level needed to produce a measurable biological response. The weight-stratified analyses in our study—showing little or no improvement among participants over 84 kg—highlight this risk [8].

From a methodological perspective, the dose–response correlation approach offers several advantages. First, it uses naturally occurring variability in exposure without needing to modify trial design. This is particularly useful in nutraceutical and botanical studies, where ethical and logistical challenges often restrict the use of multiple-dose randomized arms. Second, by analyzing exposure–response gradients, researchers can better distinguish true pharmacologic effects from high placebo noise, which has been a common issue in OA [1,15], and other conditions with large nonspecific responses to oral interventions [16]. Third, the method can be applied retrospectively to existing datasets, providing a low-cost analytical improvement that can clarify results in ambiguous trials.

More broadly, our findings reinforce essential principles of dose selection and trial design. Variability in exposure reduces statistical power, contributes to apparent differences in treatment response, and may lead to the mistaken conclusion that an intervention is ineffective. This was the fate of glucosamine and chondroitin sulfate which for years looked promising [2,3,4], but ended up losing governmental support in many countries, as it was not possible to prove that active treatment was any better than placebo. Including mg/kg analyses—as additional exploratory outcomes—can help determine the minimal effective dose and clarify the pharmacologic plausibility of observed effects—and distinguish from placebo treatment. This is especially important in botanicals, where active compounds may have low bioavailability, short half-lives, or narrow therapeutic windows.

### 4.1. Methodological Implications

The dose–response correlation approach offers several practical advantages. It is simple, transparent, and readily applied to existing datasets without the need for additional data collection or modifications to the original study design. By leveraging natural variability in body-weight-adjusted exposure, the method can help identify genuinely active botanical and pharmacological preparations in research areas where placebo responses are typically large and often obscure treatment effects. In addition, it provides a means of exploring the minimal effective dose, thereby contributing valuable information for dose selection in future clinical trials.

### 4.2. Limitations

The fact that this paper is based on a single study [8] supported by only two earlier studies [10,12] is a limitation. The method depends on sufficient variability in body weight and assumes proportional exposure with fixed dosing. Potential confounders include metabolic differences, adherence, or unmeasured pharmacokinetic variability. These limitations should be considered when interpreting dose–response correlations.

## 5. Conclusions

A simple dose–response correlation approach using body-weight-adjusted exposure revealed pharmacologic effects of *Rosa-canina* that were not apparent from conventional between-group comparisons. This method has broad applicability in any trial characterized by large placebo response and may improve identification of active preparations and dose selection in future studies. This simple dose–response correlation approach seems to be reproducible as it was demonstrated in three different studies using the same product. Additionally, it adds more insight and is low-cost and easy to perform. In the future, it might even be suggested that this approach can be included, possibly as a part of what should be available, when applying for new registrations of products.

## Data Availability

No new data were created or analyzed in this study. Data sharing is not applicable to this article.
